# Incidental Finding of a Broad Ligament Hematoma During Tubal Ligation Surgery: A Case Report and Literature Review

**DOI:** 10.7759/cureus.40120

**Published:** 2023-06-08

**Authors:** Sowjanya Kurakula, Vandana Muralidharan, Navya N, Abhijna Rao Kompella, Gayathri B K B

**Affiliations:** 1 Obstetrics and Gynecology, Mamta Institute of Medical Sciences, Khammam, IND; 2 Obstetrics and Gynecology, Sekgoma Memorial Hospital, Serowe, BWA; 3 Obstetrics and Gynecology, Nyangabgwe Referral Hospital, Francistown, BWA; 4 Obstetrics and Gynecology, Gandhi Medical College, Musheerabad, IND; 5 Obstetrics and Gynecology, Lifeline Medical Associates, New Jersey, USA; 6 Obstetrics and Gynecology, Rainbow Children’s Hospital and Birthright, Bengaluru, IND; 7 Obstetrics and Gynecology, Srinivasa Maternity and Nursing Home, Hyderabad, IND; 8 Obstetrics and Gynecology, Gayathri Bhargav Hospital, Vijayawada, IND; 9 Obstetrics and Gynecology, Dr. Pinnamaneni Siddhartha Institute of Medical Sciences & Research Foundation, Vijayawada, IND

**Keywords:** grand multipara, unscarred uterus, broad ligament hematoma, silent rupture uterus, case report

## Abstract

A silent rupture of an unscarred uterus is a rare phenomenon. Accidental diagnosis of silent rupture during sterilization procedure in a previous vaginal delivery is rarely reported. We present a case of uterine rupture in an unscarred uterus in a 40-year-old gravida 10 para 9 with intrauterine fetal demise terminated with prostaglandin E2. She was asymptomatic and hemodynamically stable. Hemoperitoneum was observed during a tubal ligation procedure performed on the third day after the abortion. A right-sided broad ligament hematoma was noticed, and surgical treatment was initiated when the patient’s condition clinically deteriorated during the operation. Our article aims to raise obstetricians’ awareness of an important causative factor of hemoperitoneum found during postpartum tubal ligation surgery.

## Introduction

Puerperal genital hematomas are relatively uncommon causes of postpartum hemorrhage but can result in severe morbidity and even maternal death. As there are no obvious symptoms of vaginal bleeding or pain, diagnosis gets delayed. Unexplained shock out of proportion to blood loss should raise a suspicion of internal bleeding [1]. Broad ligament hematomas and retroperitoneal hematomas, which form due to injury to the uterine artery or vein, or a tear in the upper vagina, cervix, or uterus that extends into uterine or vaginal arteries, and have a higher morbidity and mortality rate [2].

Diagnostic imaging studies, including ultrasonography, computed tomography scan, and magnetic resonance imaging, show the volume of estimated blood loss along with the extent of the hematoma. Treatment is variable depending on the size, location of the hematoma, and the patient's hemodynamic stability [1]. Comprehensive treatment options for broad ligament hematomas include internal iliac artery ligation, angiographic embolization, and hysterectomy [3]. The percentage of spontaneous broad ligament hematomas that result in maternal mortality is around 40% when associated with labor [4]. Our case is unique in terms of a clinically asymptomatic rupture in an unscarred uterus diagnosed on day 3 of fetal expulsion. This patient was a case of near-miss mortality and could have lost her life if she had refused to undergo tubal ligation surgery.

## Case presentation

A 40-year-old unregistered gravida 10 para 9 with 27 weeks of pregnancy presented at the prenatal clinic, with absent fetal movements for one day, which was referred by the midwife for safe institutional delivery. The patient had no previous prenatal examinations, imaging studies, laboratory tests, or vaccinations during pregnancy. There was no significant past medical, surgical, or treatment history. The patient has had nine spontaneous vaginal deliveries in the past with uneventful outcomes.

**Figure 1:**
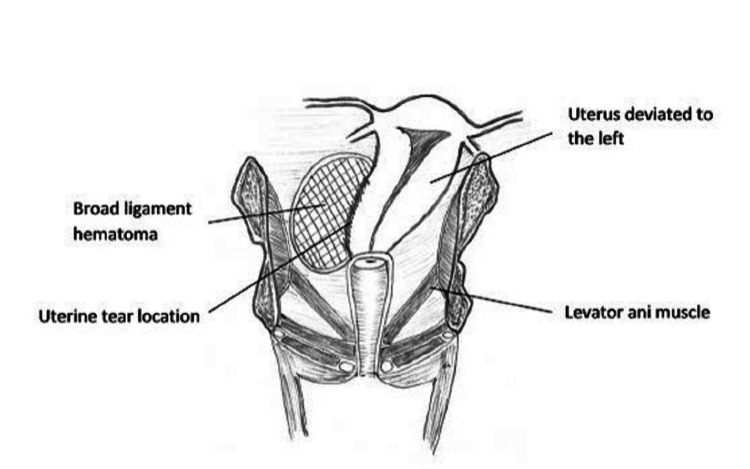
The picture shows the deviation of the uterus to the left and the location of the uterine tear. Author Sowjanya Kurakula drew the original image.

Her vital signs were within the normal range during the clinical examination. An obstetric examination revealed a pregnancy at 24-28 weeks' gestation, with no fetal heart sounds. A subsequent ultrasound examination confirmed the presence of intrauterine fetal demise. Laboratory investigations showed mild anemia. Induction of labor for termination of pregnancy with intracervical prostglandin E2 (PGE2) 0.5-mg gel was started. She expelled products of the conceptus in toto with the placenta within a few hours. The patient was administered 10 units of oxytocin intramuscular for active management of the third stage. Vital parameters were within normal limits, and clinical examination revealed a non-tender, retracted uterus below the umbilicus. Post-abortion period was uneventful, and she was transferred to the postpartum ward. The patient was motivated and convinced to undergo tubal ligation against her religious belief. She was posted for a sterilization procedure under spinal anesthesia on the third day after the abortion. Hemoperitoneum was observed during a tubal ligation procedure performed on the third day after the abortion. Intraoperative findings were as follows: right-sided broad ligament hematoma measuring 14x8 cm was noticed, the uterus deviated to the left, and approximately 500 mL of blood clots were evacuated. The uterine tear was noted in the right lateral inferior uterine segment extending vertically upwards (Figure 1).

Extensive congestion of the parietal peritoneal surface of the right lateral pelvic and abdominal wall up to the reflection of the uterovesical fold was observed during the operation. As the patient developed intraoperative hemodynamic instability due to a blood loss of more than 1,000 mL, resuscitation was performed with intravenous fluids and whole blood, and we proceeded with abdominal hysterectomy as the edges were friable, edematous, and difficult to approximate. Postoperatively, the patient received triple antibiotics with analgesic, showed good recovery, and was discharged on the fifth postoperative day. Further follow-up was conducted at the local clinic.

## Discussion

The pooled prevalence rate of uterine rupture in women with an unscarred uterus due to labor induction with PGE2 is 0.7/100,000 [5]. Our case report of stable silent uterine rupture is notable for its rare occurrence in the obstetric population.

The presence of a scar is the primary factor associated with uterine rupture, while the normal, unscarred uterus is less vulnerable to such complications. Spontaneous rupture of an unscarred uterus during pregnancy is rare and typically occurs as a result of trauma. The incidence of this condition decreases with advancements in obstetric practices. However, in developing countries, the incidence of unscarred uterine rupture remains relatively high. Obstructed labor, high parity, placenta percreta, injudicious use of uterotonic drugs, and, in rare cases, intrauterine manipulations such as internal podalic version and breech extraction are significant risk factors for unscarred uterine rupture. Numerous factors contribute to an increased risk of uterine rupture, including poor socioeconomic conditions, uncontrolled fertility, illiteracy, adolescent marriages, and underdeveloped or contracted pelvis [6]. Uterine rupture carries an additional risk when performing procedures such as laparoscopic, abdominal, and hysteroscopic myomectomies, as well as hysteroscopic septal resection [7,8].

Retroperitoneal hematomas associated with pregnancy are found more frequently in women with high parity [9]. Our case was a great-grand multipara of gravida 10 para 9 with nine living children and was at a high risk due to her age and parity. The incidence of uterine rupture was 3 in 571 grand multiparae patients regardless of the mode of delivery, and the study concluded that there is no increase in maternal morbidity and mortality in grand multiparae patients [10].

The symptomatology may vary with the rate of blood loss and the extent of the hematoma. The diagnosis of a supra levator hematoma can be delayed if the patient is asymptomatic or experiences an insidious onset of symptoms. Severe abdominal pain, abdominal heaviness, restlessness, and inability to void are some of the alarming symptoms. Pallor, hypotension, tachycardia, abdominal pain, and vaginal bleeding can be observed. Abnormal uterine displacement or irregular uterine contour per abdomen should raise suspicion of postpartum supra levator hematoma [1]. Our case was similar to that of Mendel et al., who reported two cases; a gravida 7 para 4 and a gravida 4 para 3 both presented after one day of normal vaginal delivery as a silent incomplete uterine rupture was incidentally diagnosed during postpartum tubal ligation [11].

Although magnetic resonance imaging is the most precise investigation for detecting retroperitoneal hematoma, ultrasonography is readily available, inexpensive, non-invasive, and highly sensitive [1].

Critical steps in the management of broad ligament hematoma are resuscitation, volume replacement, and surgical exploration [2]. Women with small, non-expanding hematomas and stable vital signs can be treated conservatively [12]. In our patient, a hysterectomy was considered necessary due to persistent blood loss and intraoperative hypotension. According to a study of 93 cases of uterine rupture, the majority of women in the unscarred uterus group had a tear at the junction of the upper and lower segments of the uterus along the lateral wall of the uterus. Among them, 57% of women in the unscarred uterus group and 95% in the scarred uterus group underwent uterine repair [13]. One study reported a bilateral broad ligament hematoma treated by evacuation and bilateral uterine artery ligation for a second twin delivery in a gravida 3 para 2 woman with an unscarred uterus [2]. Several treatment modalities adopted by the different authors are discussed in our study depending on available resources and circumstances (Table 1).

**Table 1:** Various clinical presentations of broad ligament hematoma in different case reports. G, gravida; P, para

Author	Year	Clinical presentation	Management approach	Maternal outcome
Mendel and Bone [11].	1956	First case: a 27-year-old, G7 P4, term gestation, diagnosed as silent rupture uterus during tubal ligation on postpartum day 1. Second case: a 36-year-old, G4 P3, term gestation, diagnosed as silent rupture uterus during tubal ligation on postpartum day 1	First case underwent a subtotal hysterectomy and Second case underwent uterine repair	Both cases recovered with no perinatal mortality
Bankada et al [2].	2015	A 30-year-old G3 P2 A0 L2 presented with second twin undelivered and obstructed labor, intraoperative finding: bilateral broad ligament hematoma	Hematoma evacuation and bilateral uterine artery ligation	Mortality- second twin
Ibrar et al [3].	2017	A 37-year-old P4 presented on a postpartum day 4 with abdominal distension and vomiting	Observation of broad ligament hematoma for spontaneous resolution; cecostomy was done for concurrent pseudo-colonic obstruction	Recovered with no perinatal mortality
Yalla et al [12].	2018	A 33-year-old G2 P1 L1, with a pregnancy of 38 weeks, presented with an acute drop in hemoglobin 3 hours after vaginal delivery	Subtotal hysterectomy with broad ligament hematoma left undisturbed as it was non-expanding	Recovered with no perinatal mortality
Bulbul et al [9].	2019	A 37-year-old, G10 P8, presented 3 days after vaginal delivery with hypovolemic shock and abdominal pain	Exploratory laparotomy revealed a giant retroperitoneal hematoma and a 3.5-cm rupture of the internal iliac artery. Also, a defect in the internal iliac vein at the level of bifurcation is noted. Both vessels ligated.	Maternal death
Bhansakarya et al [14]	2019	A 22-year-old P1 presented 7 hours after vaginal delivery with the maternal collapse	Exploratory laparotomy with ligation of bleeders.	Recovered with no perinatal mortality
Varvoutis et al [4].	2021	A 22-year-old with a pregnancy of 41 weeks and 2 days, preeclampsia, and chorioamnionitis presented to a tertiary care center after postpartum day 5 with broad ligament hematoma and sepsis	Supracervical hysterectomy	Recovered with no perinatal mortality

## Conclusions

Grand multipara has an inherent weakness in uterine musculature, making it more susceptible to uterine rupture with induction despite the careful choice of medications. Slow and less active medications would be preferable. Thorough monitoring in the postpartum period, with vital signs and complete blood count, would be helpful. Regular assessment after vaginal delivery, including vital signs recording, physical examination, and visual inspection of the genital tract, with a high index of suspicion of postpartum complications, still plays an important role in the early detection of uterine rupture. Even mild signs of shock or an increase in abdominal pain should be checked with caution. The use of diagnostic modalities such as ultrasonography and magnetic resonance imaging would help us identify the problem and early interference would reduce the morbidity. To prevent such incidents, we suggest performing a clinical bimanual examination and pelvic imaging before postpartum tubal ligation surgery.
